# Use of Capsule Small Bowel Transit Time to Determine the Optimal Enteroscopy Approach

**DOI:** 10.4021/gr404w

**Published:** 2012-03-20

**Authors:** Brandon Chalazan, Christopher J Gostout, Louis M Wong Kee Song, Felicity T Enders, Elizabeth Rajan

**Affiliations:** a Developmental Endoscopy Unit, Division of Gastroenterology and Hepatology, Mayo Clinic College of Medicine, 200 First St. SW, Rochester, USA; b Health Sciences Research, Mayo Clinic College of Medicine, 200 First St. SW, Rochester, USA

**Keywords:** Capsule endoscopy, Enteroscopy, Capsule small bowel transit time, Push enteroscopy, Single balloon enteroscopy, Double balloon enteroscopy

## Abstract

**Background:**

Capsule small bowel transit time (SBTT) is used to select the most effective enteroscopy approach when targeting capsule endoscopy (CE) findings. Aim of this study was to determine if capsule SBTT can be used to guide the choice of enteroscopy technique for reaching CE abnormalities.

**Methods:**

Single center, retrospective study involving 60 patients. Data were abstracted from medical records of patients with abnormal CE who proceeded to enteroscopy which included push enteroscopy (PE) single balloon enteroscopy (SBE) and double balloon enteroscopy (DBE).

**Results:**

Ninety five findings were documented on CE with presumed identification of 56 (59%) of these abnormalities by enteroscopy. Majority were angioectasias on CE (42%) and enteroscopy (59%). Optimal cutoff values for selection of enteroscopy procedure were: 0-21% SBTT for PE (80% sensitivity, 74% specificity, 83% PPV); 0 - 36% SBTT for antegrade SBE (93% sensitivity, 40% specificity, 82% PPV); 0 - 57% SBTT for antegrade DBE (75% sensitivity, 80% specificity, 75% PPV); and 74 - 100% SBTT for retrograde DBE (88% sensitivity, 78% specificity, 78% PPV).

**Conclusion:**

Capsule SBTT may be used to guide the selection of enteroscopy approach. PE, antegrade SBE, antegrade DBE and retrograde DBE are optimal when abnormalities on CE are seen at ≤ 21%, ≤ 36%, ≤ 57% and ≥ 74% SBTT respectively.

## Introduction

Technological advances in capsule endoscopy (CE) have resulted in enhanced visualization of the small bowel mucosa but the ability to localize abnormalities seen on CE within the small bowel remains a limitation. Although capsule small bowel transit time (SBTT) is used in clinical practice to select the most effective approach with enteroscopy (i.e. type of procedure and insertion route) when targeting CE findings, this approach has not been evaluated. Choosing the optimal approach particularly when more than one enteroscopy technique is available can be challenging.

Capsule endoscopy and the use of enteroscopy provide complementary methods for evaluating suspected small bowel pathology in particular obscure gastrointestinal bleeding (OGIB). Capsule endoscopy is a safe, non-invasive and generally well tolerated procedure with a reported diagnostic yield of 42 - 74% in patients with OGIB [1-4]. Deep enteroscopy (e.g. single balloon enteroscopy, double balloon enteroscopy) allows for tissue acquisition, improved visualization and therapeutic intervention [5-7]. The selection of the initial enteroscopy approach is important to reduce the time and number of endoscopic examinations. When CE is performed prior to enteroscopy, SBTT is typically used as a guide to select the best enteroscopic modality (e.g. SBE versus DBE) and insertion route (antegrade versus retrograde). However, the appropriate utilization of capsule SBTT has not been adequately studied. Previous studies have primarily focused on the use of CE and double DBE reporting a cutoff of 50 - 66% of SBTT as the most accurate value for selection of an antegrade DBE examination [8-11].

However, many practices have more than a single modality for small bowel enteroscopy and the initial selection of an enteroscopy approach and insertion route that provides the highest potential yield is important.

The aim of this study was to determine if capsule SBTT can be used to guide the choice of insertion route and method of enteroscopy for reaching significant abnormalities visualized on CE.

## Methods

### Patients

A retrospective analysis of patients who underwent CE and enteroscopy at Mayo Clinic, Rochester, Minnesota, from September 2006 to June 2009 was performed. Study approval was obtained from the Mayo Clinic Institutional Review Board. The patients’ electronic medical records, CE studies and endoscopic reports were reviewed. Clinical information, demographics, CE and enteroscopy data and images were abstracted. Patients provided informed consent prior to the endoscopic procedures. Enteroscopy consisted of PE, SBE and/or DBE. Only patients with an abnormal small bowel exam on CE who subsequently underwent an enteroscopy within one year were included. Patients were excluded if the CE exam was incomplete or negative for small bowel pathology, endoscopic capsule placement was required or a history of prior small bowel surgical anastomosis or resection was present. Patients with findings considered to be of doubtful clinical significance (e.g. red spots, erythema, lymphangiectasia, focal erosion, xanthoma and flecks of blood) were also excluded. Focal erosions and flecks of blood were excluded to avoid false negative enteroscopy exams as it is our experience that these CE findings are often non-specific and enteroscopy may well reach this segment of small bowel without any obvious abnormality detected.

### Capsule endoscopy

The video capsule PillCam SB or SB2 (Given Imaging, Yokneam, Israel) was used in this study. Patients underwent CE after a 12 hour fast with clear liquids allowed up to 4 hours before the test. Information on the type of small bowel finding, time to finding, duration of finding and SBTT were recorded. SBTT was defined as the time interval between capsule entry into the duodenal bulb to capsule exit into the cecum. The time from duodenal entry to finding the lesion is expressed as a percentage of SBTT (% SBTT). For the purposes of this study the following assumptions were made with CE: 1. If the same type of abnormality (e.g. angioectasia) was visualized on multiple frames for a duration of ≤ 10 mins, it was considered to be the same lesion and 2. If blood was seen over a segment of small bowel then the average time between the start and end of visualization of the blood was considered the time to finding, given that the bleeding site maybe anyway along this segment.

### Enteroscopy

PE was performed using a dedicated variable stiffness pediatric colonoscope (Olympus America, Center Valley, PA) without the use of an overtube. The SBE system (Olympus America, Center Valley, PA) consists of a flexible overtube with a distally attached inflatable balloon, dedicated enteroscope and balloon pump controller. The DBE system (Fujinon Inc, Saitama City, Japan) utilizes an enteroscope and overtube, each with an inflatable balloon mounted on the tip, and a pump controller to control both balloons. Polyethylene glycol preparation (2 - 4 liters) was used prior to all retrograde and most antegrade deep enteroscopic examinations. Antegrade exams were performed either with PE, SBE or DBE while it was our practice to preferentially use DBE for retrograde exams. Spiral enteroscopy (Spirus Medical Inc, Boston, MA) was not studied as this is a procedure not performed in our practice. The typical endpoint for enteroscopy was maximal insertion with the exception of actively bleeding pathology identified in patients with OGIB. For the purposes of this study the following assumptions were made with enteroscopy which was considered to have reached the CE small bowel abnormality if: 1. The same finding was identified on both enteroscopy and CE or 2. Enteroscopy demonstrated a vascular lesion i.e. angioectasia or Dieulafoy lesion and CE indentified blood only. A direct comparison between each technique to reach the lesion identified by CE was not performed as only few patients (17) underwent more than one procedure. The relative invasive nature of enteroscopy precludes direct comparisons between various techniques in individual patients.

### Statistical analysis

Patient characteristics were summarized with median and range or number and percent and compared between those with and without abnormalities with the Wilcoxon rank sum test or Fisher’s exact test as appropriate. We used the time from first duodenal image on the CE test to the time at which a positive finding was noted during the CE; this time was summarized as a percentage of the full SBTT (first duodenal image to first cecal image). This percentage served as the continuous predictor in a ROC curve which used the results from one of the standard tests as a gold standard: PE, SBE antegrade, DBE antegrade, or DBE retrograde. This method allowed us to assess the sensitivity and specificity of the CE test at each percentage of the SBTT for each gold standard endoscopy method. In order to minimize the chance that the same finding was repeated, we excluded CE findings within 10 minutes of each initial finding. ROC results were reported with sensitivity, specificity, positive predictive value (PPV) and area under the curve (AUC). The optimal cutoff was chosen to maximize the SBTT range as well as sensitivity and specificity.

## Results

This was a single center study. From a total of 827 patients who underwent CE during the study period, 60 patients (M = 32) met study criteria. The median age was 71.5 years (range: 25 - 87) with a median BMI of 25.8 kg/m^2^ (range: 16.8 - 39.3). There was no significant difference in median age and BMI or gender distribution between the enteroscopy groups of PE, SBE or DBE. The median SBTT (hr:min) was 3:59 (range: 1:09 - 7:33). The mean time from CE to enteroscopy was 91.6 days for PE, 54.3 days for SBE and 112.4 days for DBE. The longer time to performing DBE was primarily due to limited access and availability of this procedure. PE, SBE and DBE were performed in 32, 14 and 22 patients respectively (Table 1). A total of 77 enteroscopy procedures were performed of which 35 were PE, 14 were SBE and 28 were DBE (17 antegrade, 11 retrograde). The presumed maximal insertion point for PE was proximal jejunum, SBE was mid to distal jejunum (n = 9) and proximal ileum (n = 5), antegrade DBE was mid to distal jejunum (n = 6) and proximal to distal ileum (n = 11), and for retrograde DBE was mid to proximal ileum (n = 11).

**Table 1:** Enteroscopy Procedures

Procedures	Patients	Procedures	Finding
PE	32	35	25
SBE	14	14	15
DBE	22	28	16
TOTAL	60	77	56

PE: Push Enteroscopy, SBE: Single Balloon Enteroscopy, DBE: Double Balloon Enteroscopy.

**Figure 1:**
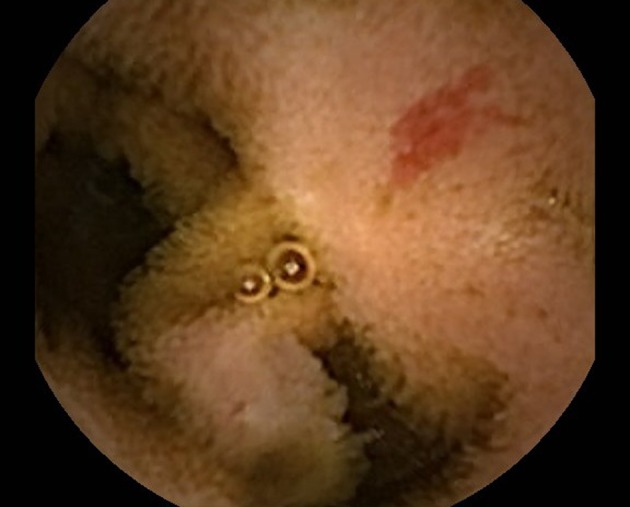
Angioectasia seen on Capsule Endoscopy.

The indication for CE in the majority of patients was OGIB (88%, n = 53). Thirty seven (70%) of these patients presented with occult OGIB. Other indications included evaluation for small bowel Crohn’s, mass, ulcer or celiac disease. Ninety five abnormal findings were documented on CE with presumed identification of 56 (59%) of these abnormalities by enteroscopy. The majority of findings were angioectasias on both CE (42%) and enteroscopy (59%) (Figure 1). Other findings (CE/enteroscopy) consisted of blood (21%/4%), ulcers (12%/7%), polyps (9%/3%), submucosal lesions (8%/11%), scalloping or fissuring (6%/11%), and miscellaneous (2%/5%). Of the 20 patients who had blood on CE, enteroscopy showed angioectasias, Dieulafoy lesion, blood and no findings in 11, 1, 1 and 7 patients respectively. There was no significant difference in gender, age and BMI (P = 0.60, 0.96, and 0.79) between those with and without abnormalities reached by enteroscopy.

**Figure 2:**
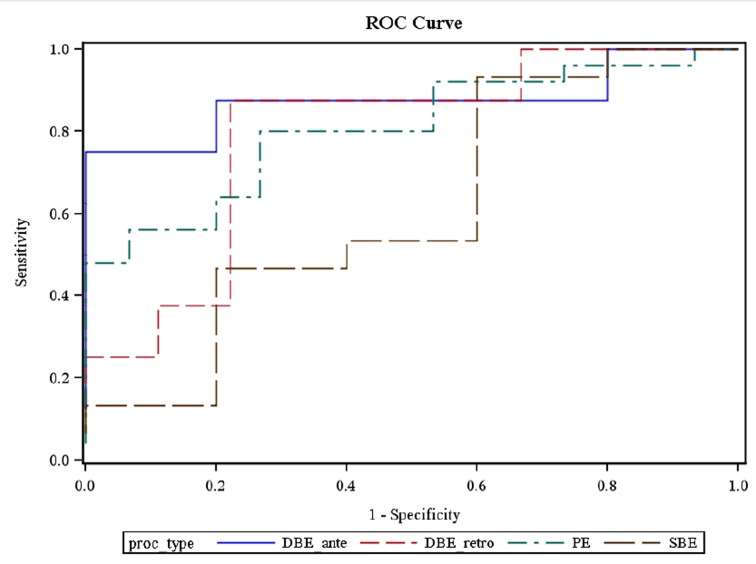
ROC curve showing the optimal cutoff values for the selection of a particular enteroscopy procedure using capsule small bowel transit time.

Using a receiver operating characteristic (ROC) curve, the optimal cutoff values for the selection of a particular enteroscopy procedure were: 0 - 21% SBTT for PE (80% sensitivity, 74% specificity, 83% PPV, 81% AUC); 0 - 36% SBTT for antegrade SBE (93% sensitivity, 40% specificity, 82% PPV, 61% AUC); 0 - 57% SBTT for antegrade DBE (75% sensitivity, 80% specificity, 75% PPV, 88% AUC); and 74 - 100% SBTT for retrograde DBE (88% sensitivity, 78% specificity, 78% PPV, 79% AUC). The likelihood of the studied enteroscopy procedures to reach and confirm CE abnormal findings between 58 - 73% SBTT was as low as 38% (Figure 2).

## Discussion

In our study the indication for CE in the majority of patients was OGIB. 59% of abnormal findings documented on CE were presumed to have been reached by enteroscopy. The majority of findings were angioectasias on both CE and enteroscopy. Based on our findings, PE, antegrade SBE, antegrade DBE and retrograde DBE are best utilized when abnormalities on CE are seen at ≤ 21%, ≤ 36%, ≤ 57% and ≥ 74% SBTT respectively.

The complementary roles of CE and enteroscopy have had a major impact on the diagnostic and therapeutic capabilities of these techniques for patients with small bowel disease [12-14]. The limitations of CE include the inability for histologic sampling, lack of therapeutic intervention and incomplete visualization of the entire small bowel in some cases. The shortfalls of enteroscopy include the invasiveness of the procedure and the difficulty in achieving total enteroscopy in all patients. The reported success rates for total enteroscopy with DBE varies from 8% to 86% with lower rates more evident in North American and European studies [15-18]. The reasons for this in part are related to the differing patient characteristics and the constraints of time spent performing enteroscopy in our practices.

The initial selection of an enteroscopy approach that provides the highest potential yield should theoretically allow for rapid diagnosis, reduction in the number and duration of procedures, improved patient compliance and cost savings. SBTT is often used to guide the choice of enteroscopy. Previous studies on SBTT have focused primarily on DBE with a lack of data on the interpretation of SBTT in other methods of enteroscopy. Nakamura et al reported a cutoff value for the selection of insertion route for DBE as 50% of SBTT [8]. Our study shows a similar cutoff of ≤ 57% for antegrade DBE but differs with regard to the retrograde approach. Other studies have suggested two-thirds of the SBTT as an indicator to choose antegrade DBE [10, 11]. Again our study shows a similar cutoff for the retrograde approach of ≥ 74% but differs for the antegrade exam. Gay et al used 75% of total transit time from capsule ingestion to cecum as the arbitrary cutoff for antegrade DBE [19]. In our study, although total enteroscopy was achieved in both our patients who had a combined DBE approach, reaching the mid segment of bowel with either approach continues to be a challenge in our patient population. SBTT is less helpful in patients with incomplete small bowel transit. As in our study, the major indication for CE and enteroscopy continues to be OGIB with angioectasia being the most common finding [20, 21].

This was a retrospective study with a small sample size and ideally the findings of this study should be reproduced in a larger prospective study. The other limitations of this study include the assumptions that 1. The capsule travels through the small bowel at a constant velocity 2. Abnormalities seen on CE that were not identified by enteroscopy were not reached rather than missed or related to self-limiting lesions such as NSAID induced ulceration 3. The abnormality identified on both procedures equated to the same lesion.

Location of intestinal lesions remains one of the challenging issues for endoscopic examinations of the small bowel. Improving localization of abnormalities seen on CE should enable more effective selection of enteroscopic procedures for lesion confirmation and therapy. Many practices have differing methods of enteroscopy available and information on the use of SBTT with various enteroscopy techniques should prove clinically beneficial. Based on our study, capsule SBTT may be used to identify the optimal enteroscopy approach. We recommend utilizing PE, antegrade SBE, antegrade DBE and retrograde DBE when abnormalities on CE are seen at ≤ 21%, ≤ 36%, ≤ 57% and ≥ 74% SBTT respectively. Reaching the mid-small bowel (58 - 73% SBTT) via deep enteroscopy remains a challenge and may require alternative radiologic or surgical intervention. Ideally future studies should be prospective and incorporate a direct comparison between the techniques soon after performance of CE but this would pose challenges, both practical and ethical, given the invasive nature of enteroscopy and the lack of ready availability of these techniques.
